# A review of the mechanism of succinylation in cancer

**DOI:** 10.1097/MD.0000000000031493

**Published:** 2022-11-11

**Authors:** Keer Lu, Dongwei Han

**Affiliations:** a Department of Prescription Science, Heilongjiang University of Chinese Medicine, Harbin, Heilongjiang, China.

**Keywords:** antineoplastic, cancer, desuccinylase, succinyl donor, succinylation, succinyltransferase, tumorigenesis

## Abstract

Lysine succinylation is a novel, broad-spectrum, dynamic, non-enzymatic protein post-translational modification (PTM). Succinylation is essential for the regulation of protein function and control of various signaling and regulatory pathways. It is involved in several life activities, including glucose metabolism, amino acid metabolism, fatty acid metabolism, ketone body synthesis, and reactive oxygen species clearance, by regulating protease activity and gene expression. The level of succinylation is mainly regulated by succinyl donor, succinyltransferase, and desuccinylase. Many studies have confirmed that succinylation plays a role in tumorigenesis by creating tissue heterogeneity, and can promote or inhibit various cancers via the regulation of different substrate targets or signaling pathways. The mechanism of action of some antineoplastic drugs is related to succinylation. To better understand the role of succinylation modification in cancer development and treatment, the present study reviewed the current research content and latest progress of succinylation modification in cancer, which might provide a new direction and target for the prevention and treatment of cancer.

## 1. Introduction

Protein modification refers to chemical modification after protein biosynthesis, also known as post-translational modification (PTM). It is generally considered to be a degradation mechanism of protein destruction or turnover to maintain physiological homeostasis. Different modifications, combinations, and changes in sites can cause alterations in the function and properties of proteins, resulting in different effects on cells.^[1]^ There Increasing evidence suggests that histone PTMs are essential for regulating various biological processes, including cell differentiation and tissue development. Furthermore, abnormal histone modification can lead to a variety of diseases, including cancer.^[2,3]^ Lysine is not only an essential amino acid for protein structure and function, but also 1 of the 3 basic residues of protein structure and function. Lysine side chain modification results in high complexity of PTM networks.^[4]^ Among the amino acids that make up proteins, lysine is the site with the most frequent post-translational modifications. Currently identified post-translational modifications of lysine sites include acetylation, propionylation, butanylation, malonylation, and succinylation.^[5,6]^ Succinylation is a novel lysine acylation reaction that has been discovered in recent years. Its proteome has been identified in Escherichia coli, Saccharomyces cerevisiae, Toxoplasma gondii, Vibrio parahaemolyticus, Mycobacterium tuberculosis, human cells, and mouse liver tissues.^[7–10]^ Studies have shown that succinylation can participate in multiple biological activities by regulating protease activity and gene expression.^[11]^ Abnormal succinylation can affect the development of several diseases such as tumors, cardiometabolic diseases, liver metabolic diseases, and nervous system diseases.^[12]^ Numerous studies have shown that succinylation modulators can promote or inhibit a variety of cancers by modulating the succinylation level of substrate targets. It also plays a regulatory role in the development of various cancers.

### 1.1. Basic characteristics andfunctions of succinylation

Succinylation is a process in which a succinyl donor covalently binds a succinyl group (-co-CH2-CH2-Co2h) to a lysine residue through enzymatic or non-enzymatic methods.^[13]^ Succinylation was first observed in Escherichia coli in 2004 and subsequently in eukaryotes.^[14]^ as a common PTM in prokaryotes and eukaryotes.^[15,16]^ In the cytoplasm, succinylation mainly occurs in the mitochondria, and is a key process of energy generation in the mitochondria.^[17,18]^ Recent studies have proven that succinylation can also occur outside the mitochondria.^[19]^ In the nucleus, succinylation occurs in more than 1-third of nucleosomes at both histone and non-histone lysine residues. Succinylation sites in the nucleus were mainly concentrated in the promoter region, suggesting that succinylation may be involved in the transcriptional regulation of genes.^[20]^ As the only respiratory enzyme involved in both the tricarboxylic acid cycle and electron transport chain, Mitochondrial complex II (CII), also known as succinate dehydrogenase (SDH), is the only respiratory enzyme involved in both the tricarboxylic acid cycle and electron transport chain.^[17]^ Studies have shown that the formation of most tumors is related to the mutation of SDH, which can lead to succinate accumulation and succinylation of lysine, which may promote tumor development.^[17,21]^ In recent years, it has been found that succinylation and acetylation highly coincide,^[7]^ and some enzymes that regulate acetylation can also regulate succinylation.^[9]^ The synergistic effect of the 2 is vital for the determination of protein structure, protein modification, and the occurrence and development of disease. After the protein is succinylated, the charge of the lysine residues changes significantly. Moreover, the addition of higher-molecular-weight succinyl groups to protein residues can significantly change the protein structure. Therefore, succinylation has a greater impact on protein properties than methylation and acetylation.^[14]^ Succinylated donors are mainly succinyl-CoA, and research has shown that succinylation levels are positively correlated with succinyl-CoA concentration.^[22]^ As an intermediate metabolite of the tricarboxylic acid (TCA) cycle, succinyl-CoA can affect a variety of metabolic processes by changing the physicochemical properties of proteins. It can be seen that succinylation shares a crucial relationship with energy metabolism. It also participates in fatty acid synthesis, amino acid degradation, electron chain transmission, ketone body formation, the TCA cycle, and other cellular metabolic processes and plays an important role in regulating a variety of cellular metabolic pathways.^[11,23]^

## 2. Research progress of succinylation in cancer

Further studies on succinylation have indicated that succinylation levels are mainly regulated by succinyl donors, succinyltransferases, and succinylases. The succinyl donor and succinyltransferase played a positive role in succinylation regulation, whereas desuccinylase played a negative role in succinylation regulation. These 3 factors affect tumor development through the regulation of different pathways. years, respectively (Table 1).

**Table 1:** Expression, gene symbol, influence, site and regulatory factors of succinylation in tumors.

Tumour	Gene symbol	Impact in tumors	Ksucc sites	Regulatory factors of succinylation	References
Pancreatic carcinoma	GLS	Promotes cancer cell proliferation and migration	K311	Succinyl-CoA	^[26]^
Pancreatic carcinoma	14-3-3ζ、β-catenin	Promotes cancer cell proliferation, migration and invasion	-	KAT2A	^[27]^
Glioma	histone H3	Tumor cell proliferation and development	H3K79	Succinyl-CoA/KAT2A/α-KGDH	^[29]^
Gastric cancer	LDHA	Promote cancer cell invasion and metastasis	K222	CPT1A	^[31]^
Gastric cancer	S100A10	Promote cancer cell invasion and metastasis	K47	CPT1A/SIRT5	^[32]^
CCRCC	HIF1、HIF2	Promotes lipid deposition and cancer progression	-	CPT1A	^[33]^
CCRCC	AGER/IL20RB/SAA1	Immune cell infiltration and m6A methylation	HNRNP (A2B1/C/G), LRPPRC/EIF3B	CPT1A/SIRT5/SIRT7/KAT2A	^[34]^
Liver cancer	PGAM1、histone H3	Promote tumor development	K99、K122	HAT1	^[36]^
Breast cancer	GLS	Promote tumor development	K146、K158	SIRT5	^[40]^
Colon cancer	PKM2	Regulation of macrophages in malignant transformation of colon cancer	K311	SIRT5	^[43]^
Lung cancer	PKM2	Promotes the proliferation of cancer cell	K498	SIRT5	^[45]^
RCC	ACOX1	It affects ROS and DNA damage response, thus affecting liver cancer development	-	SIRT5	^[47]^
CCRCC	SDHA	SIRT5 promotes CCRCC development by inhibiting succinylation of SDHA	K547	SIRT5	^[48]^
Osteosarcoma	SHMT2	Inhibits the proliferation of cancer cells	K280	SIRT5	^[51]^
Lung cancer	SOD1	Inhibits cancer cell growth	K123	SIRT5	^[52]^
Colon cancer	CS	Promotes cancer cell proliferation and migration	K393、K395	SIRT5	^[53]^

ACOX1 = acyl-CoA oxidase 1, CCRCC = clear cell renal cell carcinoma, CS = citrate synthetase, EIF3B = eukaryotic initiation factor 3 b, GLS = glutaminase, HIF1,HIF2 = hypoxia-inducible factor 1,hypoxia-inducible factor 2, HNRNP = heterogeneous nuclear ribonucleoprotein A1, IL20RB = IL-20 receptor-β, LDHA = LACTIC dehydrogenase, LRPPRC = leucine-rich penticopeptide rich domain containing protein, PGAM1 = phosphoglycerate mutase 1, PKM2 = pyruvate kinase M2, RCC = renal cell carcinoma, S100A10 = S100 calcium binding protein A1, SAA1 = serum amyloid A1, SDHA = succinate dehydrogenase complex subunit A, SHMT2 = mitochondrial serine hydroxymethyltransferase 2, SOD1 = superoxide dismutase 1.

### 2.1. Succinyl donors and cancer

As the main succinyl group donor, succinyl-CoA can be produced either through the mitochondrial membrane or in vitro. A decrease in succinyl-CoA concentration can significantly reduce succinylation.^[7]^ As a metabolic enzyme in the TCA cycle, succinyl-CoA can affect tumorigenesis by regulating cellular metabolism.^[24]^ In metabolic pathways, the balance between succinyl-CoA production and consumption may contribute to the complex regulation of succinylation and protein expression in the mediation of tumor-activated glucose metabolism, thus affecting the occurrence and development of cancer.^[25]^ Additionally, Tong et al^[26]^ demonstrated that glutaminase (GLS) can be succinylated, Succinyl-CoA synthetase adp-forming subunit β (SUCLA2) acts as an important regulator, it regulates the succinylation level of GLS K311 by regulating the concentration of succinyl-CoA. The succinylation level of GLS K311 increases when oxidative stress counteracts stress, thereby promoting the survival and proliferation of pancreatic ductal adenocarcinoma tumor cells. Therefore, changes in succinyl-CoA concentration can affect the development of tumors by regulating tumor metabolism or by acting as a substrate to regulate the level of succinyl-modification.

### 2.2. Succinyltransferase and cancer

#### 2.2.1. KAT2A and cancer.

Lysine acetyltransferase 2A (KAT2A) is a succinyltransferase whose histone succinyltransferase activity has a significant effect on tumorigenesis. Research has shown that KAT2A is highly expressed in human PDAC specimens and is positively correlated with the survival rate of patients with advanced PDAC. Additionally, its mediated histone succinylation promotes tumor cell deterioration by regulating gene expression and β-catenin stability.^[27]^ The α-ketoglutarate dehydrogenase complex was synthesized by E1k[α-ketoglutarate dehydrogenase(KGDH)(EC1.2.4.2)], E2k[dihydrolipoyl succinyltransferase (EC 2.3.1.61)], and E3k[dihydrolipoyl dehydrogenase (EC 1.8.1.4)].^[28]^ α-Ketoglutarate dehydrogenase complex can act as a trans-succinylase, mediating succinylation in an α-ketoglutarate-dependent manner, directly or indirectly regulating succinyl-CoA levels to maintain adequate levels of succinylation, succinylate various mitochondrial proteins, and alter their function.^[16]^ Stu succinylation dies have revealed that KAT2A can act as an α-KGDH-dependent histone succinyltransferase and bind to α-KGDH to promote the succinylation of H3K79 in tumor cells and promote the proliferation of glioma cells.^[29]^ It can be seen that histone succinylation can cause a wide range of gene expression changes, thus promoting tumor growth.

#### 2.2.2 CAPT1A and cancer.

Kurmi et al^[30]^ found that carnitine palmityl transferase (CPT1A) exhibits lysine succinyltransferase (LSTase) activity in vivo and in vitro. As a type of LSTase, CPT1A can regulate the enzyme activity and metabolism of substrate proteins independent of its classical carnitine palmityl transferase (CPTase) activity. Recently, researchers have studied the impact of CAPT1A on gastric cancer pathogenesis. Studies have confirmed that CAPT1A mediates succinylation of LACTIC dehydrogenase (LDHA) at the K222 site, which can inhibit the degradation of LDHA by reducing the interaction between LDHA and selective autophagy adaptor protein 1 (SQSTM1), thus promoting the invasion, metastasis, and growth of gastric cancer cells.^[31]^ Furthermore, CPT1A can also regulate the succinylation of S100 calcium-binding protein A1 at the K47 site and inhibit its ubiquitin-dependent protease degradation, thus leading to the accumulation of S100 calcium binding protein A1 in gastric cancer cells and promoting the development of gastric cancer.^[32]^ In a study on renal clear cell carcinoma (CCRCC), CPT1A was found to exhibit a lower expression level and significantly lower level of activity in the mitochondria harvested from tumor tissue than in adjacent normal kidney tissue. Direct inhibition of CPT1A by hypoxia-inducible factor 1 and hypoxia-inducible factor 2 leads to a decrease in fatty acids entering the mitochondria, forcing fatty acids to form lipid droplets for storage, thus promoting the progression of CCRCC.^[33]^ Succinylation regulators may also affect the progression of CCRCC by regulating tumor immunity and m6A methylation regulators. CPT1A and SIRT5 can upregulate the expression of leucine-rich penticopeptide rich domain containing protein and eukaryotic initiation factor 3 b, respectively, and regulate CCRCC pathogenesis.^[34]^

### 2.2.3 HAT1 and cancer

Histone acetyltransferase 1(HAT1) is a type B histone acetyltransferase that regulates the acetylation of histones and non-histones.^[35]^ Existing studies have shown that HAT1 regulates the level of lysine succinylation in a variety of proteins, participates in many cellular physiological and pathological pathways, and increases significantly in multiple tumor tissues. It is considered to play the role of succinyltransferase in histones and non-histones with respect to tumorigenesis.^[36]^ For histones, HAT1 is a novel histone succinyltransferase that catalyzes histone H3K122 succinylation to enhance epigenetic regulation and gene expression profiling. For non-histones, HAT1 catalyzes the succylation of the glycolytic enzyme phosphoglycerate mutase 1 on K99 in tumor cells, resulting in increased enzymatic activity and stimulation of glycolysis flux in cancer cells.

### 2.3. Desuccinylase and cancer

COBb was the first desuccinylase discovered in prokaryotes, with both deacetylation and desuccinylation activities.^[4]^ SIRT5 and SIRT7 are desuccinylases in eukaryotes. Currently, a considerable number of studies have been conducted on SIRT5. As a member of the sirtuin family, SIRT5 can reverse the level of succinylation,^[37]^ and its abnormal expression is related to carcinogenesis. GLS is often up-regulated during tumorigenesis.^[38]^ Research has demonstrated that SIRT5 can regulate glutamine metabolism through the desuccinylation of GLS and protect GLS from ubiquitin-mediated degradation, thus affecting the occurrence and development of human breast tumors.^[39]^ Additionally, research has shown that BAG3 can promote the succinylation of GLS at Lys158 and Lys164 by downregulating SIRT5 expression and interacting with mitochondrial GLS, inhibiting proteasome degradation, and stabilizing GLS, thus promoting glutamine metabolism, activating autophagy, and affecting the proliferation of tumor cells.^[40]^

Pyruvate kinase M2 (PKM2), an important metabolic kinase in the Warburg effect of tumor cells, regulates the final step of glycolysis to ensure an adequate energy supply to the cells.^[41]^ Increasingly, studies have proven that PKM2 is overexpressed in various cancers and has a negative effect on the proliferation of tumor cells, affecting various biological processes of tumor cells.^[42]^ Studies have shown that SIRT5 interacts directly with PKM2. PKM2 has varying succinylation sites in different tumor cells. For instance, succinylation of the K311 site plays an important role in regulating the enzymatic activity of PKM2, while SIRT5 desuccinylation and activation of PKM2 play a vital role in the regulation of macrophage metabolism in colitis and colon cancer.^[43]^ After glucose starvation, lysine succinylation at position 433 of PKM2 increased the migration of PKM2 to the mitochondria and enhanced its interaction with mitochondrial outer membrane voltage-dependent anion channels (VDAC3) in human colon cancer cells. PKM2 stabilizes VDAC3 by inhibiting its ubiquitination and degradation of VDAC3, thereby increasing mitochondrial permeability and ATP production, thus promoting cell survival and tumorigenesis.^[44]^ The Regulation of SIRT5 on PKM2 has also been observed in lung cancer cells. SIRT5-mediated desuccinylation of PKM2 at K498 enabled it to maintain an antioxidant response, thus supporting the survival and proliferation of cells under acute oxidative stress. SIRT5 inhibition hinders the proliferation of lung cancer cells through desuccinylation at this site.^[45]^

The SDH complex is composed of multiple subunits including SDHA, SDHB, SDHC, and SDHD. Mutations in succinate dehydrogenase complex subunit A (SDHA) can cause the loss of SDH enzyme activity in tumor tissues, resulting in the accumulation of succinate acid, which can cause pseudohypoxia and lead to increased angiogenesis and other SDHX gene mutations. SDHA’s abnormal expression of SDHA plays a key role in tumorigenesis.^[46]^ In HCC samples, it was found that the stable downregulation of SDHA increased the succinylation level of endogenous ACOX1, which promoted the dimerization of ACOX1. SIRT5 negatively regulated the activity of ACOX1 by inhibiting formation of the active ACOX1 homologous dimer. Thus, ROS and DNA damage responses are affected and the occurrence and development of HCC are regulated.^[47]^ SDHA, which is closely related to succinylation level, can also interact with SIRT5 directly. It was found in the tissues of CCRCC that SIRT5 mediates the desuccinylation of SDHA at K547, and silencing SIRT5 can lead to the super-succinylation and reactivation of SDHA, thus affecting the occurrence and development of CCRCC.^[48]^

Mitochondrial serine hydroxymethyltransferase 2(SHMT2) is a key enzyme in single-carbon unit metabolism that is significantly elevated in most cancer types.^[49]^ It has been reported that SHMT2 can promote the proliferation of cancer cells by catalyzing the rate-limiting step of serine catabolism.^[50]^ SIRT5 mediates the desuccinylation of Lys280 to SHMT2, activates the enzyme SHMT2, and promotes serine catabolism in tumor cells.^[51]^

Moreover, SIRT5 has been proven to affect tumor development by regulating the expression levels of superoxide dismutase 1(SOD1) and citrate synthase (CS). SIRT5 can bind desuccinate and activate SOD1. When SIRT5 is co-expressed, SOD1-mediated ROS production is accelerated and mutated at the K123 site, which inhibits the proliferation of lung tumor cells.^[52]^ It has been reported that SIRT5 interacts with CS. SIRT5 desuccinylates CS at K393 and K395, Supersuccinylation of those sites significantly decrease their enzyme activity, thereby inhibiting the development of colon cancer.^[53]^ Additionally, human RIDA (hRIDA) has been proven to be a carcinogenic antigen, and its expression is negatively correlated with tumor differentiation.^[54,55]^ Studies indicate that hRIDA is expressed in most cell lines and is regulated by lysine succinylation, which is negatively correlated with the cell proliferation rate and regulated by SIRT5.^[56]^

### 2.4. Other factors that affect cancer development through succinylation modification

In addition to the influence of the key regulatory factors of succinylation, succinylation modification also affects the occurrence and development of tumors through other pathways. Lysine succinylation and acetylation have been reported to occur at the same site in different tissues^[7]^ which can affect the function of proteins and their target genes without changing the gene sequence and creating a second genetic code favorable to carcinogenesis. For example, the levels of succinylation and acetylation increase simultaneously in breast cancer tissues, and the 2 may be co-regulated by members of the histone deacetylase family.^[57]^ Nucleophosmin 1(NPM1) is the only protein in which acetylation and succinylation occur at the same lysine site and is highly conserved in many distinct species. The expression of NPM1 is upregulated in breast cancer, and the acetylation and succinylation of NPM1 may lead to a DNA damage response by regulating the chromosome structure, thus affecting the development of breast cancer.^[58]^

In addition, succinylation can regulate the occurrence and development of neoplasms through the pentose phosphate pathway and endoplasmic reticulum (ER) protein processing pathways.^[59]^ For example, studies on the quantitative proteome and succinyl group of breast cancer found that the succinylation modification levels of BiP, GRP94, and CRT proteins in TK and ER processing of pentose phosphate pathway were significantly upregulated, and these molecular chaperones and folding enzymes were closely related to the progression of cancer.^[60,61]^ Even under aerobic conditions, tumor cells obtain the energy necessary for survival through glycolysis, a phenomenon known as the Warburg effect.^[62]^ In an overall quantitative study of the proteome and related protein lysine succinylation in renal cell carcinoma tissues, the upregulation of succinylated proteins was found to be rich in metabolically related processes. These processes include glycolysis and amino acid biosynthesis, suggesting that lysine succinylation is closely related to metabolic regulation in renal carcinoma cells. Renal cell carcinoma progression is closely related to the glycolysis pathway, suggesting that lysine succinylation plays an important role in energy metabolism.^[63]^

## 3. Succinylationand antineoplastic drugs

Succinylation has been proven to be related to the mechanism of action of some antitumor drugs. For instance, after intestinal cancer cells were treated with sodium dichloroacetate, 179 protein succinylation sites were upregulated, and 114 protein succinylation sites were downregulated.^[64]^ Subsequently, 34 post-translational modification sites of histones, including H4K20suc, were detected for the first time in bladder cancer cells treated with the HSP90 inhibitor AUY922.^[65]^

Succinylation also plays a positive role in radioimmunotherapy. Moreover, succinylation of streptavidin (STAV) has been reported to significantly reduce renal accumulation by generating highly negative surface charges without affecting its affinity to biotin.^[66]^ In pre-targeted radioimmunotherapy, the treatment strategy of biotinylated bevacizumab and succinylated STAV can accelerate blood clearance and lower renal reabsorption, resulting in reduced tumor progression and may be effective for treating triple-negative breast cancer.^[67]^ Succinylation of the single-chain antibody cc49-STAV structure may significantly reduce the renal dose by inhibiting the reuptake of fusion proteins in proximal renal tubules, which can improve the therapeutic index related to the multi-step immune targeting method of radioimmunotherapy.^[68]^

Mechanism-based cancer treatment strategies have been proposed in recent years. For example, pharmacological inhibition of SIRT5 inhibited tumor growth in breast cancer mice, and the mice were not shown to be toxic.^[69]^ Pharmacological inhibition or enhancement of succinylation regulatory enzyme activity may become a new direction for tumor growth inhibition. Furthermore, inhibition of hypersuccinylation is also considered a way to intervene in tumors associated with high succinylation. Studies have shown that *R*-2-hydroxyglutaric acid generated by the NADP(+)-IDH mutation induces mitochondrial supersuccinylation, thus inducing cancer metabolism and apoptotic resistance, and that removing supersuccinylation can inhibit the tumorigenic growth of cells containing the IDH mutation.^[70]^

## 4. Conclusions

Lysine succinylation, a newly discovered post-translational modification of proteins, is involved in various biological processes and participates in the development of various tumors. Although there are many studies on the regulatory mechanisms of succinylation in various neoplastic diseases, only a limited number of systematic reviews are available in this field. Therefore, this article provides a summary of succinylation and its correlation with different control factors in the regulatory mechanism of tumorigenesis and its application in anticancer drugs. In order to provide a comprehensive and systematic summary, focus must be placed on amber acylation and its role in the development of tumors to provide new ideas for clinical diagnosis. It provides potential directions for further understanding the mechanism of antitumor drugs and the development of new antitumor drugs. Moreover, as a new research topic, succinylation modification has many undiscovered mysteries worth exploring; for instance, the role played by multiple modifying enzymes in carcinogenesis, the synergistic role played by the multiple modification functions of enzymes in tumorigenesis, determining whether other undiscovered factors play a synergistic role in addition to the discovered regulatory factors of succinylation, the relationship between histone and non-histone of various enzymes regulating succinylation modification, the relationship between the functions of various catalytic active enzymes, and the potential mechanism of succinylation regulating signaling pathways. However, the application of the role played by succinylation in tumorigenesis in pharmacological studies is mostly at the level of in vitro experiments, lacking further experimental verification.

## Author contributions

**Data curation:** Keer Lu.

**Project administration:** Dongwei Han.

**Supervision:** Dongwei Han.

**Writing – review and editing:** Keer Lu,Dongwei Han.
